# Personalized Medicine: Recent Progress in Cancer Therapy

**DOI:** 10.3390/cancers12041009

**Published:** 2020-04-19

**Authors:** Valentina Gambardella, Noelia Tarazona, Juan Miguel Cejalvo, Pasquale Lombardi, Marisol Huerta, Susana Roselló, Tania Fleitas, Desamparados Roda, Andres Cervantes

**Affiliations:** 1 Department of Medical Oncology, INCLIVA Biomedical Research Institute, University of Valencia, 46010 Valencia, Spain; vgambardella@incliva.es (V.G.); noetalla@incliva.es (N.T.); jmcejalvo@incliva.es (J.M.C.); mhuerta@incliva.es (M.H.); srosello@incliva.es (S.R.); tfleitas@incliva.es (T.F.); 2 Instituto de Salud Carlos III, CIBERONC, 28220 Madrid, Spain; 3 Department of Oncology, University of Turin; Candiolo Cancer Institute - FPO- IRCCS, 10060 Candiolo (TO), Italy; pasquale.lombardi@ircc.it

**Keywords:** precision medicine, personalized medicine, translational oncology, new drug development

## Abstract

Translational research has revolutionized how we develop new treatments for cancer patients. The change from an organ-centric concept guiding treatment choice towards deep molecular analysis, driving a personalized approach, is one of the most important advances of modern oncology. Several tools such as next generation sequencing and RNA sequencing have greatly improved the capacity to detect predictive and prognostic molecular alterations. Detection of gene mutations, amplifications, and fusions has therefore altered the history of several diseases in both a localized and metastatic setting. This shift in perspective, in which attention is focused on the specific molecular alterations of the tumor, has opened the door to personalized treatment. This situation is reflected in the increasing number of basket trials selecting specific molecular targets. Nonetheless, some weaknesses need to be addressed. The complexity of cancer cells enriched with concomitant molecular alterations complicates identification of the driver. Moreover, tumor heterogeneity could be responsible for the lack of benefit when targeted agents are used. In light of this, there is growing interest in the role of multidisciplinary committees or molecular tumor boards to try to enhance selection. The aim of this review is to critically analyze the evolution of cancer treatment towards a precision approach, underlining some recent successes and unexpected failures.

## 1. The Revolution of Tumor Treatment: From Tumor Site to Molecular Alterations

During the last few years, cancer patient treatment has been completely revolutionized as several molecular alterations have been identified as drivers of cancer development and progression [1]. This biomarker-based approach has historically proceeded from basic science to validation in the clinic. Thus, to guide treatment choice, attention has gradually turned to biomarkers rather than the anatomic site of origin of a given tumor [2]. Due to this change, large groups of patients whose tumors bear a particular molecular feature have been successfully treated with a single targeted drug. The first example of molecular-based medicine for cancer patients was the use of endocrine therapy in luminal breast cancer [3]. However, the point probably marking the revolution towards a precision medicine approach was the approval of imatinib in patients with chronic myeloid leukemia bearing the t (9,22) translocation that creates a BCR-ABL fusion kinase [4].

The identification of druggable molecular alterations marked a new era in oncology [5]. The most important results were achieved in breast [6,7,8,9] and lung cancer [10,11,12] and in melanoma [13]. In breast cancer, apart from identifying hormonal receptors as therapeutic agents, other pinpointed biomarkers such as *PIK3CA* (Phosphatidylinositol 4,5-bisphosphate 3-kinase catalytic subunit alpha isoform) or *ERBB2* (human epidermal growth factor receptor 2) mutations have completely altered the therapeutic approach in luminal breast cancer patients [7,14,15,16]. Moreover, the detection of HER2 amplification as a driver has also contributed immensely to locating another important subgroup of patients who definitely benefit from anti-HER2 inhibition in all clinical settings [17,18,19]. A fundamental shift was also observed in patients diagnosed with non-small cell lung cancer (NSCLC). The identification of *EGFR* (Epidermal Growth Factor Receptor) mutations [20] and EML4-ALK (echinoderm microtubule associated protein-like 4- Anaplastic lymphoma kinase) translocation [21] has affected outcomes for advanced diseases. Moreover, identification of the *BRAF*-V600E mutation and the subsequent treatment with BRAF and MEK inhibitors [14] has significantly improved clinical outcomes in patients diagnosed with advanced melanoma. These represent only a few examples of the application of precision medicine in daily clinical practice. Indeed, the genomic revolution has impacted significantly on the majority of solid tumors nowadays, involved also in “orphan diseases” like extrahepatic cholangiocarcinoma [22].

Pinpointing specific molecular characteristics (such as microsatellite instability across all solid tumors), somatic or germline mutations, (such as *BRCA*, *BRAF* or *ERBB2*) [23,24,25,26], or gene fusions (*FGFR* and *NTRK)* [27,28] could guide treatment choice in this field, improving patient outcomes independently of tumor location. To properly apply these molecular-based treatments, it is ultimately necessary to distinguish which patient groups will probably benefit or not from this type of therapy by identifying specific biomarkers predictive of response or resistance [29].

Although genomics seems to be a really relevant start point to plan a precision approach for cancer patients, it is clear that molecular phenotype measurements and characterization are a needed arm to the understanding of tumor to improve the precision medicine approach. In some cases, the use of proteomic could also help when several molecular alterations are detectable, making it difficult to identify the most relevant driver to be targeted [30]. Moreover, genetic mutations do not always result in the predicted change of the corresponding protein, and there are many other factors that contribute to tumor behavior, such as protein modifications, metabolism, and the microbiome [31]. Another important field to try to improve a precision medicine approach is metabolomic [32]. The importance of it is not only for the identification of targetable biomarkers but also for the identification of pharmacological phenotype able to understand the mechanisms of inter-patient variability in response to drug therapy [33]. Metabolomics can also help in evaluating drug resistance and disease relapse [34,35], leading to opportunities for the development of novel therapeutic strategies [32].

**Figure 1:**
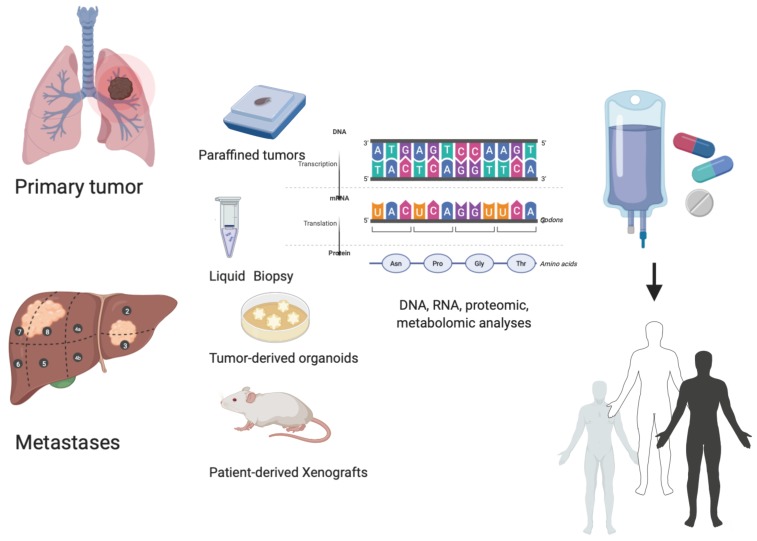
Personalized treatment: an integrated precision approach. How to personalize cancer treatment from the molecular evaluation of primary tumor or metastases evaluating liquid biopsy, tumor-derived organoids, and tumor-derived xenografts.

Identifying those molecular features has led to a clinical strategy in which testing upfront or after first-line failure may provide certain novel therapeutic opportunities. In umbrella trials, patients diagnosed with the same type of solid tumors are treated according to their molecular features, while in basket trials, patients diagnosed with different types of solid tumors with a common driver molecular alteration are selected and treated with a specific inhibitor [36]. As an example, a multisite study (including >1000 patients with non-small cell lung cancer) showed that matching was associated with longer survival than was seen in patients without genotype-directed treatment. Likewise, two meta-analyses [37,38] in 70,000 patients reported that trials with a personalized strategy led to a higher proportion of responding patients and longer progression-free and overall survival than trials with unselected patients. Standout results were obtained in patients diagnosed with tumors harboring *ERBB2* mutations or *NTRK or FGFR* fusions [15,27,28] (Figure 1).

In the MOSCATO 01 trial, cancer treatment was planned according to genomic analyses. A potentially actionable molecular alteration was identified in 411 of 843 patients. A total of 199 patients were treated with a targeted agent matched to a genomic alteration. The Progression-Free survival 2/1 (PFS2/1) ratio was >1.3 in about one-third of the patients. Although objective responses were observed in only 11% of patients, progression-free survival with this molecularly guided strategy was longer than obtained by the previous line of therapy in one-third of 194 patients. This study suggested that high-throughput genomics could improve outcomes in molecularly selected patients when treated accordingly [39].

## 2. Precision Molecular Oncology: Understanding the Role of New Drivers with Novel Drugs

Several potential targets for novel drugs have been identified using high-throughput technologies, and several compounds have recently been approved or are under investigation.

One of the most prevalent molecular alterations in solid tumors are *PIK3CA* mutations. The PI3K/AKT/mTOR (The phosphatidylinositol 3-kinase (PI3K)/AKT/mammalian target of the rapamycin (mTOR)) pathway is an intracellular signaling pathway implicated in cell proliferation. This pathway can be activated at several points, but *PIK3CA* mutations and PTEN (Phosphatase and tensin homolog) function loss are the most frequent detectable molecular alterations [40]. For this reason, several basket trials have been conducted or are still ongoing to assess the role of PIK3CA inhibitors in several solid tumors. In luminal breast cancer, incidence of *PIK3CA* mutations is about 30% in primary tumors and metastases. In a phase III trial enrolling *PIK3CA* mutant luminal breast cancer patients progressing to hormonal treatment, adding alpelisib, a specific p110α PIK3CA inhibitor, to fulvestrant significantly improved antitumor activity (response rate 26.6% vs. 12.8%) and progression-free survival (11 vs. 5.7 months) over a combination of placebo plus fulvestrant, leading to its regulatory approval. These results confirm that *PIK3CA* mutations could be drivers in metastatic luminal breast cancer and suggest the importance of detecting this molecular alteration in selected patients [7]. Other more specific novel PI3K inhibitors are currently under clinical development (Table 1).

Activation of fibroblast growth factor receptor (FGFR) pathways also plays a major role. FGFRs are made up of four highly conserved transmembrane tyrosine kinases receptors (FGFR1–4) and FGFR5, which lacks an intracellular kinase domain. All these receptors are activated by fibroblast growth factor (FGF), participating in cell survival and proliferation. Deregulation of the FGF signaling axis is implicated in oncogenesis, tumor progression, and resistance to anticancer therapy across several solid tumors, with a prevalence below 10%. Multiple trials studying diverse solid tumors have proposed the aberrant FGFR signaling pathway as a potential therapeutic target, and several inhibitors are under development. FGFR alterations may consist of amplification, mutations, and gene-fusion, yet the specific role of each one and their contribution in predicting drug response is not clear [56]. In early clinical trials, promising results were reported in patients who show *FGFR2* amplification in gastric cancer and in patients with FGFR2 and FGFR3 translocations in cholangiocarcinoma and urothelial cancers, respectively. Disappointingly, only modest levels of clinical activity have been reported for patients with other aberrations, such as *FGFR1* amplification7 or *FGFR2* mutation, in advanced-stage endometrial cancer [57]. Urothelial cancer is one of the tumors that most harbors FGFR alterations. In particular, the luminal I subtype is characterized by a lack of benefit from immunotherapy and a higher rate of mutations in the gene encoding for FGFR, detectable in about 20% of all urothelial cancers [58]. In a phase II study [59] treating patients diagnosed with locally advanced and unresectable or metastatic urothelial carcinoma with FGFR alterations, the potent FGFR1–4 tyrosine inhibitor erdafatinib demonstrated antitumor activity, achieving objective responses in 40% of previously treated patients. Interestingly, patients who had undergone previous immunotherapy also achieved the same benefit. *FGFR2* activation by point mutations and FGFR1–3 amplification or overexpression are also observed in subsets of patients with advanced cholangiocarcinoma [49]. The most common alterations are chromosomal fusions consisting of FGFR2 exons 1 to 17, encoding the intact extracellular and kinase domains, fused in-frame to a 3′ partner that possesses a protein dimerization domain. Multiple FGFR-selective inhibitors are being tested in clinical trials in cholangiocarcinoma patients harboring FGFR pathway alterations (Table 1).

BRAF mutations are major oncogenic drivers in 7% of solid tumors [60]. BRAF inhibition has led to significant improvement in melanoma. In colorectal cancer, *BRAF* mutation has been associated with specific clinicopathological features as well as very poor prognosis. *BRAF* mutations are more prevalent in proximal colon tumors, poorer differentiation, mucinous histology, and older females [61]. However, several clinical trials testing BRAF inhibitors as single agents in *BRAF*-mutant colon cancer patients saw response in less than 5%, mostly of short duration. This lack of efficacy was attributed to feedback EGFR activation resulting in MAPK (mitogen-activated protein kinase) signaling pathway reactivation. Based on this hypothesis, the combination of BRAF/MEK inhibitors plus monoclonal antibodies against EGFR has been explored. In a phase III trial, 665 metastatic colorectal patients with *BRAF-V600E* mutation who had experienced treatment failure with one or two prior regimens were recruited to receive binimetinib, encorafenib, and cetuximab versus irinotecan-based chemotherapy. This triplet therapy was well tolerated with a manageable safety profile. Moreover, it showed a better response rate than chemotherapy (26% vs. 2%) as well as significantly improved overall survival (OS) for those receiving the triple targeted agent combination (9 vs. 5.4 months), becoming a new standard of care for previously treated *BRAF V600E*–mutant metastatic colorectal cancer patients and underlining the need to uncover *BRAF* mutations in all advanced colon cancer patients [62].

**Figure 2:**
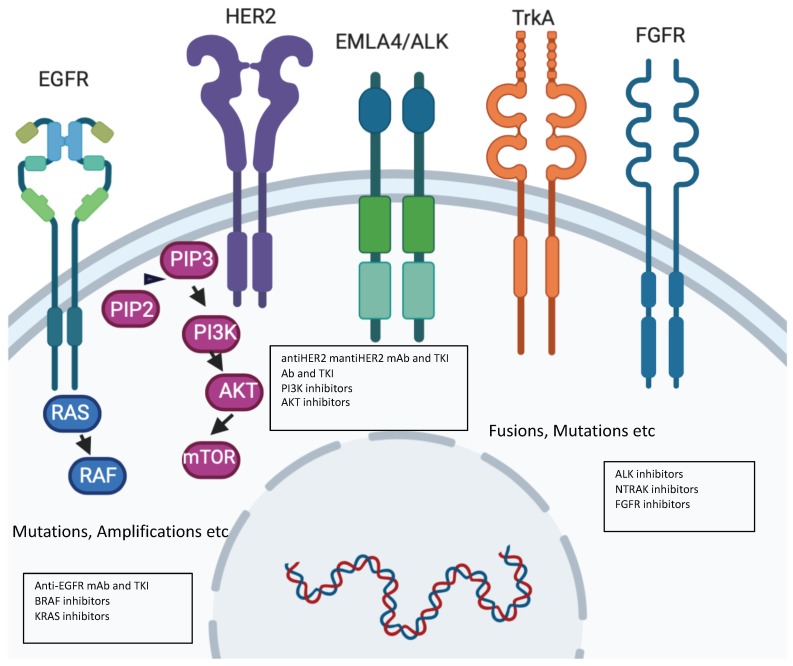
Molecular alterations driving precision oncology.

Recently, NTRK fusions have also been detected as a possible target [63]. The neurotrophic tyrosine receptor kinases (NTRK) plays a key role in nervous system development. These are three receptors, TrkA, TrkB, and TrkC, with a tyrosine kinase intracellular domain able to activate signaling pathways like MAPK, PI3K/AKT, and PLCγ/PKC (phospholipase-Cγ and protein kinase C signal pathways) resulting in enhanced migration, cell differentiation, synapse formation, and proliferation. In NTRK fusions, the 3′-region is fused with a 5′ region of a fusion partner, resulting in a chimeric receptor protein with dimerization, and thus, uncontrolled activation of the TRK kinase domain leads to chimeric proteins with constitutively activated or overexpressed kinase function conferring dominant oncogenic potential. These oncogenic fusions can be either intra or interchromosomal. All three NTRK genes are potentially affected. These alterations are considered rare, being present in about 0–25%, with the highest occurrence in secretory breast cancer, mammary analog secretory cancer (MASC) of salivary glands, congenital mesoblastic nephroma and infantile fibrosarcoma thyroid cancers, spitzoid melanomas, and gastrointestinal stroma tumors (GIST) [63]. The presence of NTRK fusion seems to indicate early alterations in solid tumors, so the use of specific inhibitors would supposedly improve clinical outcomes. Larotrectinib, a selective TRK inhibitor, was tested in adults and children whose tumors harbored a NTRK 1-2-3 fusion. The overall response rate was 75% [28]. Other NTRK inhibitors are currently under investigation, and some have shown clinical activity in patients resistant to first-generation inhibitors due to the development of secondary mutations (Table 1 and Figure 2).

## 3. Limitations of Molecular Driven Treatments in the Clinic

Despite a promising biological background justifying precision medicine, certain negative results have highlighted limitations in the molecularly driven treatment strategy. The SHIVA trial [64] could serve as a paradigm for what is still incompletely understood when applying for precision medicine in cancer patients. In this trial, 741 patients diagnosed with any kind of solid tumor were screened for molecular alterations and randomized to receive the physician in charge’s treatment of choice versus targeted therapy selected according to their molecular profile. No benefit in median PFS was observed in molecularly oriented patients versus conventional approach (Hazard-ratio (HR) 0.88, *p* = 0.41), suggesting that off-label use of molecularly targeted agents does not improve progression-free survival compared with standard treatment [64]. Nevertheless, critical analysis can reveal certain limitations potentially responsible for the failure of this approach [65]. It is well known that the presence of multiple molecular alterations together with tumor heterogeneity consistently limits the use of monotherapy, which could justify the lack of benefit derived from a precision approach [66]. Moreover, elements of the approach used in this trial are questionable, such as hormonal therapy in heavily treated patients or nonspecific inhibitors when targeting certain molecular alterations [64].

In the NCI-MATCH trial, patients diagnosed with solid tumors are randomized according to their molecular alterations in 40 different arms. In three cohorts from the NCI-MATCH trial, patients with tumors harboring *ERRB2* amplification, FGFR alterations, or *PIK3CA* mutations were treated with T-DM1, FGFR inhibitors, or taselisib, respectively. Unfortunately, response rates were very low, under 10% across several cohorts [67]. In this case, the presence of heavily pretreated populations and concomitant molecular alterations could justify the lack of benefit.

Moreover, several discordant results have been obtained using selected targets in solid tumors sharing the same molecular alterations. It is widely known for instance that blocking HER2 in breast cancer has been associated with significant improvement in clinical outcomes [3,8,19], although the use of antiHER2 agents in gastric or cancer did not confer the same results [68]. The same negative results were observed when BRAF inhibitors were used as single agent in melanoma or colorectal cancer CRC, suggesting that, aside from the molecular alterations, in these mutations, several other molecular characteristics need to be considered when applying precision medicine to cancer patients. For these reasons, tumor heterogeneity, molecular mechanisms of resistance in pretreated patients conferring a complex pattern of mutations, could cause resistance to single targeted agents and could become the final cause of the imprecision of precision medicine. Moreover, Several limitations could also derive from pharmacodynamic or kinetic aspects that could cause inter- and intraindividual variability, limiting the use of targeted agents such as gender, weight, ethnicity, and renal and hepatic functions [69]. A precision approach could be difficultly adopted when a patient presents more than one factor which could interfere with drug exposure or response. [70]. Therefore, an implementation of precision medicine should be researched. The preliminary results from the NCI-MATCH trial highlight a critical biological reality that has been known for some time: genomic alterations do not always lead to oncogenic pathway activation or addiction, and targeting multiple drivers and/or resistance pathways may be required for optimal antitumor efficacy [5,71,72]. A relevant limitation of targeted therapies is the presence of molecular alterations responsible for primary or acquired resistance [68]. This phenomenon has been widely studied and described in all solid tumors. The amplification of transmembrane receptors or intracellular proteins hyperactivation among the same pathway to which the targeted agent is driven or among other cellular pathways implicated in cancer cell proliferation and survival could be responsible for the lack of response to a specific inhibitor [73].

### Limitations of the Molecular Approach

Another point that should be addressed is the evolution of the specific molecular tools used for the selection of patients enrolled in the clinical trials. In some precision medicine-based trials, patients were screened by immunohistochemistry (IHC) or PCR to evaluate the presence of a protein expression or hotspots mutation. However, nowadays, the use of a complete Next Generation Sequencing (NGS) panel able to evaluate a wide screen of molecular alterations is quite common. The analysis performed by NGS or RNA sequencing provides high sensitivity in the detection of specific molecular alterations and also the capability of detecting concomitant alterations that could cause eventually resistance to specific targeted agents [64,74] (Table 2).

A better understanding of cancer biology is needed to optimize and define context-dependent oncogenic mutations and resistance mechanisms. In this scenario, preclinical models, such as patient-derived xenografts and organoids, may help elucidate potential codrivers and resistance mechanisms so that rational combinations can be designed and tested to support clinical deployment [81]. Analyses of circulating tumor DNA may also provide insights regarding dynamic changes that correspond to drug response or resistance, as has been observed for instance with RAS mutations in patients with colorectal cancer treated with epidermal growth factor receptor inhibitors [72].

## 4. How to Overcome Limitations: Functional Precision Medicine, Liquid Biopsy, and Molecular Tumor Board

### 4.1. Functional Precision Medicine: The Role of Patient-Derived Organoids (PDOs) and Patient-Derived Xenografts in a Personalized Approach

Functional precision cancer medicine has the potential to complement current genomic approaches. The minor role of molecular profiling in predicting response to targeted therapies and limitations of preclinical models currently used for drug selection have hindered correct validation of precision medicine [82]. The principal objective of functional models is the possibility to dynamically evaluate cancer evolution and clonal selection due to exposure to an anticancer drug. These models help us to grasp possible causes of treatment failure. During the last few years, 3D culture (the so-called organoids) and patient-derived xenograft models have both facilitated functional analysis for drug efficacy in solid tumors. Patient-derived xenograft (PDX) models have contributed in implementing translational medicine. These models are derived from the transplantation of patient tumor cells into immunodeficient mice. These models were found to reproduce in a more similar way the original tumor characteristics versus previous models in vivo. PDXs conserve the original tumor characteristics preserving the heterogeneity. PDXs could help in predicting drug sensitivity and resistance in several tumor types, being a good tool to improve personalized approach [83,84].

Derived from patient tumor cells, the organoids preserve tissue biological characteristics better than the more commonly used monolayer cell cultures. The possibility to test directly on the tumor of a specific patient represents a great step towards a personalized approach. In an intriguing recently published experiment, the PDOs analyzed showed 100% sensitivity, 93% specificity, 88% positive predictive value, and 100% negative predictive value in predicting patients’ response to treatment (Fisher’s exact test *p* < 0.0001). This marked a major breakthrough in precision medicine, suggesting that PDOs can contribute to treatment choice [85].

### 4.2. Dynamic Evaluation of Tumors: The Role of Liquid Biopsy

Another promising tool that has greatly facilitated dynamic evaluation of solid tumors is plasma cell-free DNA (cfDNA) analysis by liquid biopsy. Although a specific targetable biomarker can be identified, resistance always appears. For this reason, evaluation of tumor heterogeneity and clonal selection due to treatment pressure need to be deeply characterized. Several series have suggested that cfDNA could evaluate tumor heterogeneity by detecting the molecular mechanisms of acquired resistance [71,86]. This analysis allows genomics and other molecular alterations to be assessed at a specific moment, leading to the accurate evaluation of tumor development. In acquired resistance, the evolutionary pressure of therapy can drive outgrowth of distinct tumor sub-clones harboring independent resistance mechanisms within an individual patient, within different metastatic lesions, or even within the same lesion [87]. Moreover, the genomic analysis of a single biopsy upon disease progression may play a primary role in identifying mechanisms of acquired resistance. Nevertheless, this single-biopsy approach might not capture the massive heterogeneity of cancer cell populations shaped by the selective pressures of target therapy. cfDNA may offer advantages for assessing tumor heterogeneity. cfDNA analysis can potentially identify multiple concurrent heterogeneous resistance mechanisms that a single-biopsy approach could miss. In a recently published work, direct comparison of cfDNA versus tumor biopsy in the setting of acquired resistance illustrates how single-lesion tumor biopsies frequently fail to identify the presence of multiple clinically relevant resistance mechanisms, with cfDNA identifying additional concurrent resistance mechanisms in 78% of cases [88].

### 4.3. Molecular Tumor Board: Why Do We Need It?

The past few decades have seen a critical improvement in cancer treatment outcomes through a combination of molecular tumor testing and genetically matched targeted therapies. The history of precision medicine can be dated back to the first use of antiestrogen in breast cancer. Since this breakthrough, several other predictive biomarkers have been identified. Currently, the number of somatic tumor mutations used for standard treatment decisions is still quite limited and can easily be assessed via platforms capturing only these mutations, yet the demand for genetic testing evaluating molecular alterations will expand proportionally to rapidly expanding genetic knowledge and related drug development. Clinicians will be confronted with both increasingly complex genetic information and a wide array of platform choices; although large-scale sequencing is most informative in most cases, targeted in-depth sequencing may be preferred in others. The abundance of genetic tests and information provides a serious challenge. Furthermore, most pathology and sequencing reports only document well-known aberrations that could be therapeutically targeted, meaning that other genetic aberrations for which experimental agents and/or drug access programs are available may be missed [89]. Therefore, as novel molecular and genomic treatment indications are explored, it is becoming increasingly vital to generate and correctly interpret molecular tumor profiles to offer optimal cancer treatment. Nevertheless, the remarkable evolution of molecular techniques and the precision achieved by these tools need skilled assessment. This requires discussing the clinical cases and results obtained by molecular analyses to translate molecular profiles into clinical benefit for our patients. Developing molecular tumor boards made up of experts from a variety of disciplines will help optimize patient selection [90].

## 5. Conclusions

Personalized treatment for patients diagnosed with solid tumors has resulted in several advances in recent years. The possibility to offer a molecular-based personalized approach for cancer patients represents an attractive possibility in oncology. To obtain a relevant and real change which could improve all clinical outcome, a better understanding of molecular biology is needed. To improve, a multi-omic approach able to integrate DNA and RNA alterations, proteomics, and metabolomics will be necessary. The implementation of translational studies based on liquid biopsy and organoids or xenografts to evaluate molecular changes due to clonal pressure generated due to the use of target agents or tumor heterogeneity would help in the detection of mechanisms of resistance, suggesting the possibility for novel combinations. Further research is urgently needed to improve precision.

## Figures and Tables

**Table 1:** Novel targeted agents against PIK3CA^1^, FGFR^2^, and NTRK^3^ molecular alterations under development for solid tumors in phase I trials.

Class of Inhibitors	Novel Targeted Agents under Development for Solid Tumors in Phase I Trials		
	Compound Name	Mechanism of Action	Phase
PI3K inhibitors	GDC0077 [41]	Potent PI3K alpha inhibitor	Ib
	MEN1611 (PA799) [42]	PI3K alpha inhibitor	Ib
	AMG319 [43]	AMG319 is a PI3Kδ inhibitor. Preclinically, target inhibition abrogates Treg-mediated immunosuppression, augmenting CD8+ T-cell antitumor activity	IIa
	CH5132799 [44]	Oral pan-PI3 kinase inhibitor	Ia/b
FGFR inhibitors	AZD4547 [45]	Potent and selective inhibitor of FGFR 1, 2, and 3	I
	NVP-BGJ398 [46]	Oral, selective, ATP-competitive inhibitor of FGFR1, 2, and 3	I
	E-7090 [47]	Oral and selective inhibitor of FGFR1, 2, and 3	I
	LY2874455 [48]	Inhibitor of FGFR 1, 2, 3, and 4	I
	TAS-120 [49]	Potent and highly specific against wildtype FGFR1–4 as well as against some FGFR2 kinase domain mutations	I
	BLU-554 [50]	Potent and selective inhibitor of FGFR4	I
	H3B-6527 [51]	Selective and covalent inhibitor of FGFR4	I
	FGF-401 [52]	Potent and selective, reversible-covalent small-molecule inhibitor of FGFR4	I
NTRK inhibitor	LOXO-195 [53]	Selective inhibitor of TRK	I
	TSR-011 [54]	Dual ALK^4^ and TRK inhibitor	I
	DS-6051b [55]	Inhibitor with high affinity for ROS1 ^5^ and TRK	I

**^1^** PIK3CA = Phosphatidylinositol-4,5-Bisphosphate 3-Kinase Catalytic Subunit Alpha; FGFR ^2^ = Fibroblast Growth Factor Receptor; NTRK ^3^ = Neurotrophic tropomyosin receptor kinas; ALK ^4^ = Anaplastic lymphoma kinase; ROS1 ^5^ = c-ros oncogene 1.

**Table 2:** Molecular tools for selecting patients in clinical trials.

Molecular Tools for Selecting Patients in Precision Medicine-Based Basket Trials	
Clinical Trial	Molecular Tools
Bisgrove [75]	Immunohistochemistry, Fluorescence in situ hybridization microarray
IMPACT [76]	PCR-based genomics and NGS
SHIVA [64]	Targeted NGS-based
MOSCATO [39]	Targeted NGS-based, RNA Seq
MyPathway [77]	Genomic testing
Profiler [78]	Targeted NGS-based
I-PREDICT [79]	Targeted NGS-based, ctDNA
WINTHER [80]	Targeted NGS-based, Transcriptomic
